# From Insulating PMMA Polymer to Conjugated Double Bond Behavior: Green Chemistry as a Novel Approach to Fabricate Small Band Gap Polymers

**DOI:** 10.3390/polym9110626

**Published:** 2017-11-16

**Authors:** Shujahadeen B. Aziz, Omed Gh. Abdullah, Ahang M. Hussein, Hameed M. Ahmed

**Affiliations:** 1 Advanced Polymeric Materials Research Laboratory, Department of Physics, College of Science, University of Sulaimani, Qlyasan Street, Sulaymaniyah 46001, Kurdistan Regional Government, Iraq; omed.abdullah@univsul.edu.iq (O.G.A.); ahang.hussein@univsul.edu.iq (A.M.H.); hameed.ahmad@univsul.edu.iq (H.M.A.); 2 Development Center for Research and Training (DCRT), University of Human Development, Qrga Street, Sulaymaniyah 46001, Kurdistan Regional Government, Iraq

**Keywords:** dye doped polymer, extract GT solution, FTIR study, band gap analysis, Urbach energy, XRD study

## Abstract

Dye-doped polymer films of Poly(methyl methacrylate) PMMA have been prepared with the use of the conventional solution cast technique. Natural dye has been extracted from environmentally friendly material of green tea (GT) leaves. Obvious Fourier transform infrared (FTIR) spectra for the GT extract were observed, showing absorption bands at 3401 cm^−1^, 1628 cm^−1^, and 1029 cm^−1^, corresponding to O–H/N–H, C=O, and C–O groups, respectively. The shift and decrease in the intensity of the FTIR bands in the doped PMMA sample have been investigated to confirm the complex formation between the GT dye and PMMA polymer. Different types of electronic transition could be seen in the absorption spectra of the dye-doped samples. For the PMMA sample incorporated with 28 mL of GT dye, distinguishable intense peak around 670 nm appeared, which opens new frontiers in the green chemistry field that are particularly suitable for laser technology and optoelectronic applications. The main result of this study showed that the doping of the PMMA polymer with green tea dye exhibited a strong absorption peak around 670 nm in the visible range. The absorption edge was found to be shifted towards the lower photon energy for the doped samples. Optical dielectric loss and Tauc’s model were used to estimate the optical band gaps of the samples and to specify the transition types between the valence band (VB) and conduction band (CB), respectively. A small band gap of around 2.6 eV for the dye-doped PMMA films was observed. From the scientific and engineering viewpoints, this topic has been found to be very important and relevant. The amorphous nature of the doped samples was found and ascribed to the increase of Urbach energy. The Urbach energy has been correlated to the analysis of X-ray diffraction (XRD) to display the structure-properties relationships.

## 1. Introduction

Polymer materials are broadly used in photonic device fabrication. Dye-doped polymers have grown to be very popular for their diverse advantages. Moreover, they can be used in linear and nonlinear photonic devices [1]. Recent studies reveal that lasers created out of such dye-doped polymers have several applications in sophisticated nanoscale lasers, optical telecommunication devices, and novel chip-integrated photonic biosensors [2]. The dye-doped polymers are also known as unique photoconverters. Based on their structure, they can possibly absorb and emit light in the visible and near-infrared (NIR) regions of the electromagnetic spectrum [3]. Poly (methyl methacrylate) (PMMA) is a high-strength commercially available amorphous thermoplastic polymer. PMMA exhibits prominent mechanical, dimensional, and thermal stabilities, as well as a high optical transparency with a relatively low glass transition temperature [4,5]. PMMA is resistant and stable to acid and alkaline media, owing to its rigid behaviour [6]. It is well reported that the optical characterizations of solid polymer films are crucial to obtain knowledge regarding their energy gap, refractive index, and dielectric constant, which are vital for various optical applications [7]. The prepared dye-doped polymeric materials that exhibit suitable optical properties are found to be promising candidates for the applications of solar cells, photonic devices, optical fibres, laser media, and electronic sensors [8,9]. The natural and synthetic dyes are compounds of great interest as they play a significant role in our everyday life [10]. The dye-doped polymers are considered to be potential materials in optoelectronics particularly in making devices, employment in organic light emitting diodes (OLED), liquid crystal (LC) displays, quantum electronics, electroluminescence, solar cells, and energy storage [10,11]. Triphenylmethane, azo, anthraquinone, perylene, and indigoid dyes are more interesting among the large number of dye categories [10].

Several dye-doped polymers were reported in previous studies. A maximum absorption peak at around 564 nm for the doped PMMA polymer with a well-known rhodamine B/chloranilic acid (Rho B/CHA) has been observed in [8]. They have achieved a bandgap of 3.1 eV after γ-irradiation. Hamdy et al. [6] have used methylene blue (MB) as a doping dye material and a distinguishable peak at around 654 nm has been achieved in their study. Sun et al. [12] have studied the phenanthrenequinone (PQ)-doped PMMA as a photopolymer material for fast response in optoelectronics applications. In photonic networks, fast uncomplicated and economical fabrication process are required to achieve a successful application of solid-state dye lasers that can reliably produce a large number of lasers with tunable wavelengths, configuring at almost any time [2]. In this study, a natural dye, which is extracted from green tea (GT) leaves, was used as a doping dye. It is well known that tea derived from *Camellia sinensis* leaves is the most widely consumed drink globally. It can be classified, in accordance with the level of oxidation, into three major types: green (unoxidized), oolong (partially oxidized), and black (fully oxidized) tea [13]. Previous studies confirmed from the high pressure liquid chromatography (HPLC) observations that theanine, theobromine, gallic acid, gallocatechin, caffeine, epigallocatechin, catechin, epicatechin, epigallocatechingallate, gallocatechingallate, epicatechingallate, and catechingallate are the major components of GT extracts [13,14], which contains a very large number of OH/NH functional groups and their conjugated double bonds. Thus, the dye of green tea holds many conjugated and functional groups, which are found to be considerably important in the dye-doped polymer preparation. The intensive and extensive survey of previous studies reveals that absorption peaks at high wavelength cannot be exhibited from most of the dye-doped polymers. The primary objective of the present study is to fabricate a dye-doped polymer with an absorption peak at high wavelength, using a natural dye obtained from environmentally friendly materials. The results can also provide more knowledge in the field of dye-doped polymers. To the best of our knowledge, our findings reveal the suitability of dye-doped PMMA polymer for photonics and solar cell applications due to its small band gap.

## 2. Experimental

### 2.1. Preparation of Dye-Doped PMMA Solid Polymeric Films

**Figure 1:**
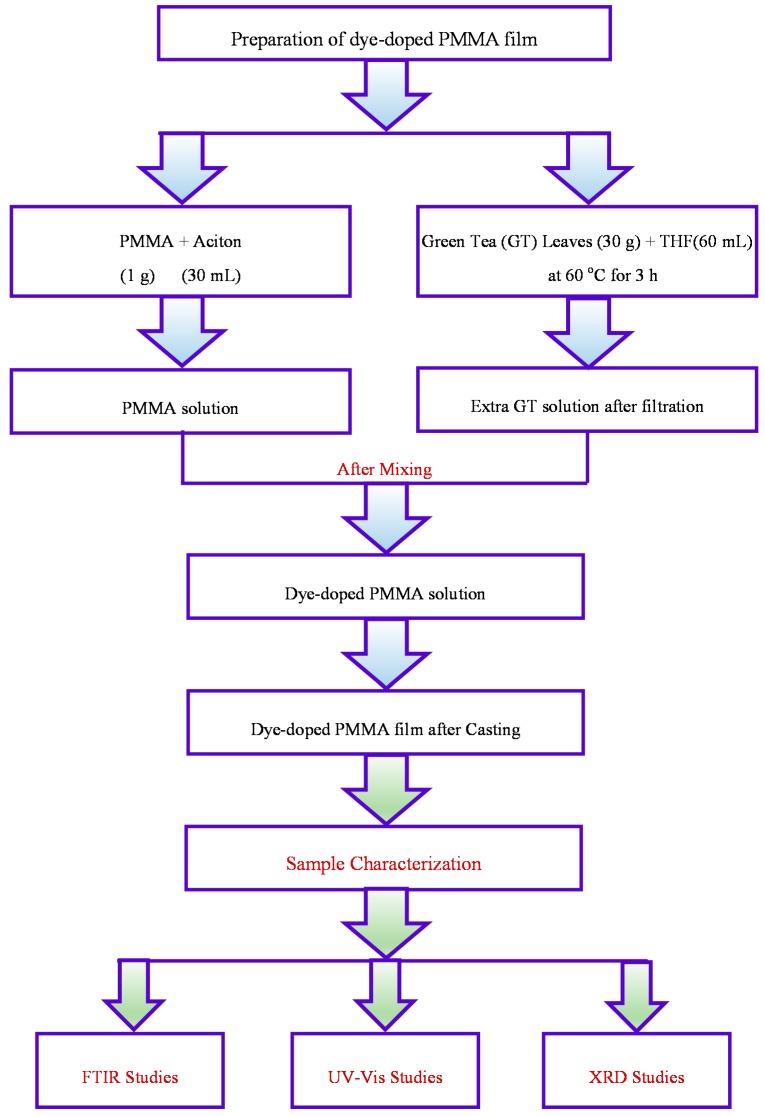
Flowchart of the experimental method and characterization techniques.

The PMMA polymeric material used in this study was supplied by Sigma-Aldrich (Saint Louis, MO, USA). The well-known solution casting technique was used to prepare the dye-doped PMMA polymer films. First, 1 g of PMMA powder was dissolved in 30 mL of acetone at room temperature. The mixture was then stirred using a magnetic stirrer for approximately 4 h. Natural colorant tea extract was derived from green tea leaves. For this purpose, 30 g of green tea leaves was added to 60 mL of tetrahydrofuran (THF) solvent at 60 °C for 3 h, without exposing the solution to direct sunlight. The solution was left to be cooled down to room temperature. Whatman filter paper (Whatman 41, cat. No. 1441, Maidstone, UK) with a pore size of 20 μm was then used to remove the residues. Then, 14 mL and 28 mL of GT extract solution were added to the homogeneous PMMA solutions and continuously stirred for 5 h. The solutions were cast into different Petri dishes and dried at room temperature to form the films. The thickness of the films ranged from 120 to 121 μm was controlled by casting the same amount of PMMA. Prior to optical characterization, the films were kept in a desiccator with blue silica gel for further drying. The samples were coded as GT 0, GT 14, and GT 28 for PMMA incorporated with 0, 14, and 28 mL of extracted GT solution, respectively. Figure 1 shows the flowchart of the experimental work undertaken.

### 2.2. UV–VIS Measurement

The optical absorption spectra of the solid polymer films have been collected using an ultraviolet–visible near-infrared (UV–VIS–NIR) spectrophotometer (Jasco SLM-468, Tokyo, Japan) in the absorbance mode.

### 2.3. FTIR and X-ray Diffraction Analysis

The complex formation between the GT extract and PMMA polymer was investigated using Fourier transform infrared (FTIR) spectroscopy. The FTIR spectra were collected using a Thermo Fischer Scientific (Waltham, MA, USA) Nicolet iS10 FTIR spectrophotometer in the wavenumber region 400–4000 cm^−1^ with a resolution of 2 cm^−1^. The X-ray diffraction (XRD) was recorded at room temperature using an X-ray diffractometer (NL-7602 EA PANalytical B.V., Almelo, The Netherlands) with an operating voltage and current of 40 kV and 45 mA, respectively. The samples were scanned with a monochromatic X-ray beam of wavelength λ = 1.5406 Å and glancing angles of 5° ≤ 2θ ≤ 90° with a step size of 0.05°. The required experimental techniques for sample characterization are shown in Figure 1. 

## 3. Results and Discussion

### 3.1. FTIR Study

**Figure 2:**
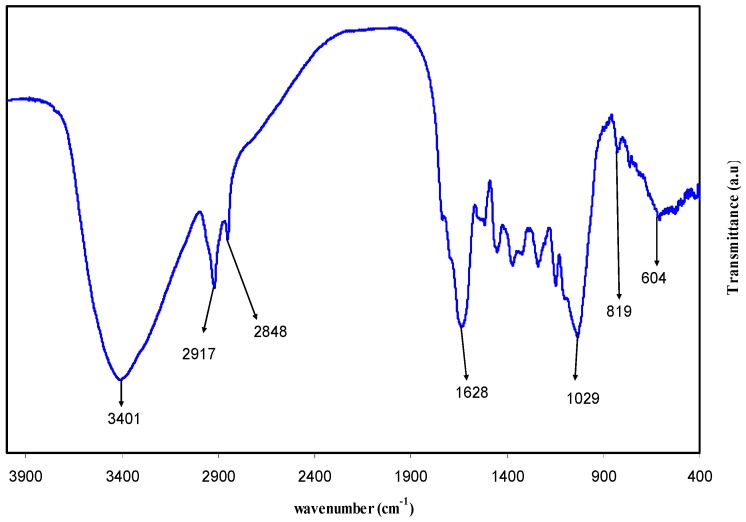
Fourier transform infrared (FTIR) spectra of pure green tea (GT) extract.

Figure 2 shows the FTIR spectrum of the GT extract solution. Recent studies have exposed great interest in the use of natural dyes. This is a result of the fact that they are recognized as being environmentally friendly, along with having other properties, such as deodorizing, being lower in toxicity, and showing anti-allergenic, anti-bacterial, and anti-cancer properties [15,16]. An intense broad band appearing at 3401 cm^−1^ is found to be attributed to the N–H and O–H stretching modes of polyphenols [17,18]. A strong band at 1628 cm^−1^ can also be assigned to the C=C stretch in the aromatic ring and the C=O stretch in polyphenols [18,19]. The C–H and O–H stretches in alkanes and carboxylic acid have been found to appear at 2917 and 2848 cm^−1^, respectively [18]. The C–O stretching in amino acid has also caused a band at 1029 cm^−1^ [18,19]. Earlier studies have established that the FTIR bands of tea extracts containing polyphenols have appeared at 3388 cm^−1^, 1636 cm^−1^, and 1039 cm^−1^, which are referred to O–H/N–H, C=C, C–O–C stretching vibrations, respectively [18,19,20,21]. Therefore, from the IR spectrum, one can observe that carboxylic acid, polyphenols, and amino acid are the main functional groups in the green tea sample.

**Figure 3:**
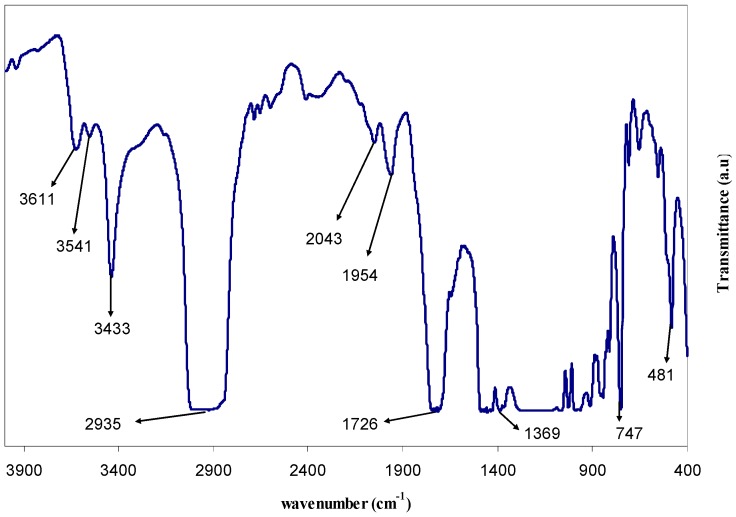
FTIR spectra of pure PMMA polymer (GT 0). It is obvious that the peaks are sharp and their intensity is high.

**Figure 4:**
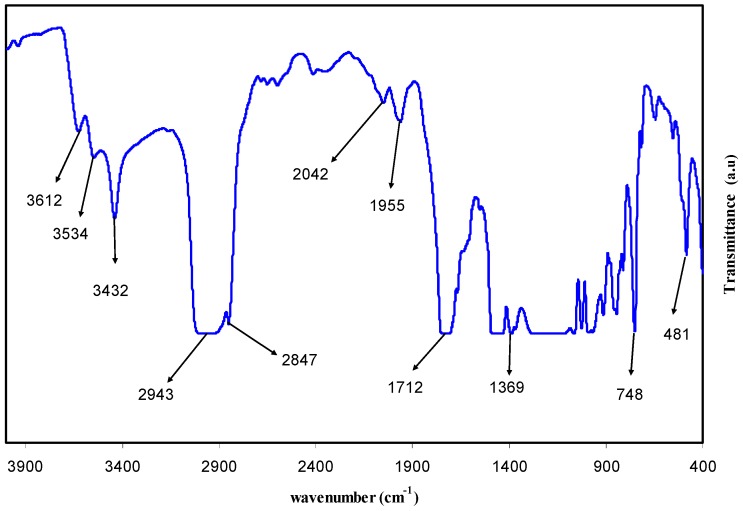
FTIR spectra of dye doped PMMA polymer (GT 28). Shifting of peaks and their broadening is evidence for the complex formation between the extract GT solution and PMMA polymer.

The FTIR spectra of pure PMMA polar polymer and PMMA doped with 28 mL of extract GT solution are shown in Figure 3 and Figure 4. FTIR spectroscopy has long been recognized as a powerful tool for the elucidation of structural information. The position, intensity, and shape of vibrational bands are useful in clarifying conformational and environmental changes of polymers at the molecular level [22]. It was well established that functional groups in organic compounds have absorptions which are characteristic not only in position, but also in intensity [23]. The strong band appearing at 1726 cm^−1^ in the spectrum (see Figure 3) of the pure PMMA sample can be attributed to the carbonyl (C=O) group [22] and shifts to 1712 cm^−1^ with lower intensity and broad character in the doped PMMA sample (see Figure 4). Thus, the shift in peak position, decrease in intensity, and broadening of the peak due to the C=O in the doped PMMA sample clearly indicates the miscibility between the PMMA and GT extract solution. The FTIR bands appearing from 950–481 cm^−1^ in Figure 3 and Figure 4 are due to the bending of C–H [24]. The peak at 2935 cm^−1^ (Figure 3) can be ascribed to –CH stretching and shifts to 2943 in the doped PMMA sample (Figure 3) and a new peak at 2847 cm^−1^ appeared, which is attributed to carboxylic acid groups of the GT extract solution (see Figure 2) [18]. The band appeared at 3433 cm^−1^ in the FTIR spectra of pure PMMA is related to the N–H stretching vibration [25,26], and shifts to 3432 cm^−1^ with a significant decrease in intensity as depicted in Figure 3. The valuable change in intensity of N–H band is an evidence for a large amount of N–H functional groups in GT extract. Additionally, the FTIR spectra of GT extract solution (see Figure 2) shows the existence of a N–H group with strong intensity at 3401 cm^−1^. Thus, the decrease in intensity of the IR band at 3432 cm can be ascribed to the complex formation between the GT extract solution and PMMA polymer. The FTIR spectrum of pure PMMA obtained in the present work is very similar to that reported by Soman and Kelkar [27]. The shifting in the FTIR bands and the decrease in intensity is evidence for the occurrence of miscibility between the PMMA polymer and the GT extract solution.

### 3.2. Absorption and Absorption Coefficient Study

**Figure 5:**
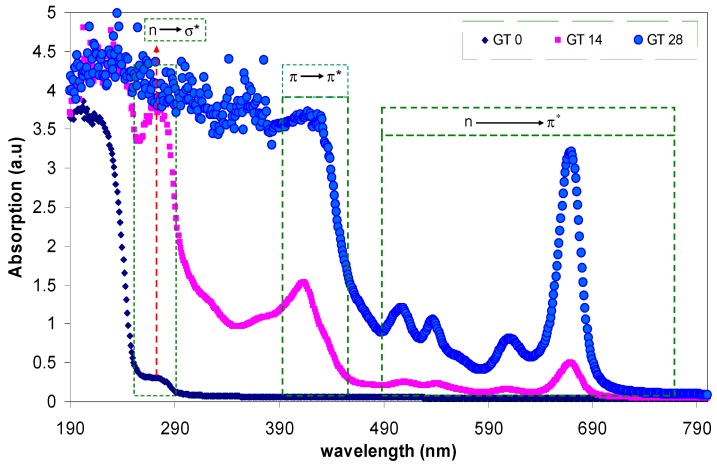
The absorption spectra of pure PMMA and PMMA doped samples.

**Figure 6:**
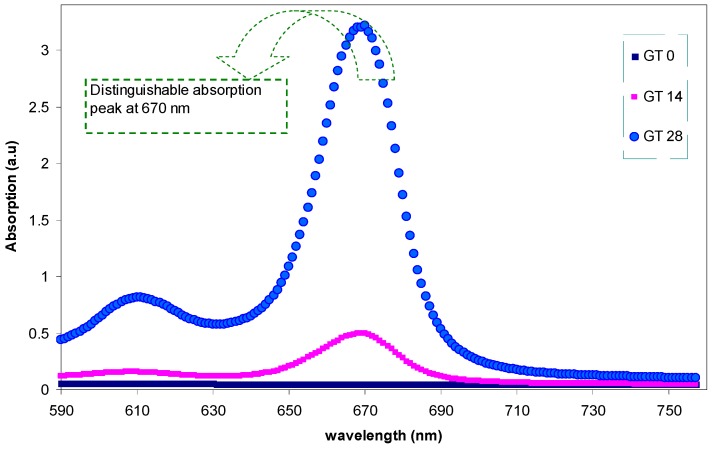
The absorption spectra of pure PMMA and PMMA doped samples at longer wavelengths.

Figure 5 shows the absorption spectra of pure PMMA and PMMA doped samples. Here, from the absorption spectra of the doped samples, it is achievable to obtain almost all of the different types of electronic transition. The absorption of light or photon energy, in the UV and visible regions, by polymeric materials involves the σ, π, and *n*-orbitals electrons to be promoted from the ground state to higher energy states that are described by molecular orbital [28]. The electronic transitions involved in the ultraviolet region, 160–260 nm, can be ascribed to *n*→σ* transition [27], while π→π* and *n*→π* transitions require relatively low energy and, hence, occur at higher wavelengths, as shown in Figure 5. The absorption peaks that were observed at high wavelengths, 400–700 nm, for the PMMA doped samples are related to the existence of π electrons [28,29,30]. Similar absorption spectra, for extracted GT in ethyl acetate solvent, have been reported [31]. It was well established that conjugated systems comprising alternating double bonds are considered to be a central class of materials for the applications of optoelectronic devices due to their π-excessive nature [32]. The shifting towards the longer wavelengths indicates the small band gaps of the doped samples [33]. It was reported that strong shifts towards the longer wavelengths can be attributed to the existence of π-delocalization along the polymer chain. This postulation is further supported by the absence of absorption peaks in absorption spectra of pure PMMA polymer [32]. The source of π-delocalization in the doped samples is found to be related to the structure of the extracted GT solution containing polyphenols, amino acids, alkaloids, proteins, glucides, minerals, volatile compounds, and trace elements [14]. Polyphenols comprise the most interesting group of GT leaf components [34]. The most determined chemicals or molecular structures of the components of the extracted GT solution can be observed elsewhere [13,14,34,35]. Earlier studies confirmed that the extracted GT solution contains adequate conjugated double bonds, hydroxyl (OH), carboxylic (C=O) groups, polyphenols, and polyphenol conjugates which are convenient for the formation of complexes with functional (polar) groups of polymeric materials [13,14,34,35,36]. The results of FTIR clearly showed the complex formation between the GT dye and PMMA polymer (see Figure 3). Dye-doped PMMA as a polymer optical waveguide has received considerable attention for its usage in optoelectronics devices and optical components, owing to its low cost and volume productivity [37]. Figure 6 shows the absorption spectra of pure PMMA and dye-doped PMMA samples at longer wavelengths. One can see from the figure that the GT 28 sample exhibits a distinct and intense peak at 670 nm, which reveals its suitability for photonics and optoelectronics applications. Utilization of natural dye is the novelty of this study in comparison to previous studies of other researchers. Furthermore, the intensity of the peak (3.460) is higher than those reported in previous studies for dye-doped PMMA polymer. Previous studies have confirmed the promising role of dye-doped polymer films for erasable/rewritable optical discs, developed by optical data systems. A considerable number of patents have reported the combinations of polymer and dye for optical data storage [38].

**Figure 7:**
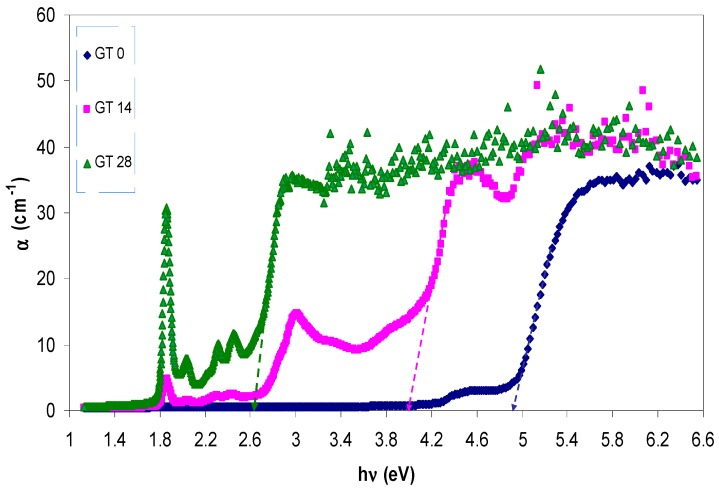
The absorption coefficients versus photon energy for pure PMMA and PMMA doped samples.

Figure 7 represents the absorption coefficient variation with photon energy for the pure and doped PMMA samples. The absorption edge investigation is found to be significant in interpreting the novel changes that occur in the electronic structure of doped materials [39]. It is obvious from the spectra that, upon addition of extracted GT solutions to the pure PMMA sample, the absorption edge are shifted towards lower photon energy sides. The absorption edge is a region in which an electron is excited, from a lower energy state to a higher energy state, by an incident photon. The optical absorption coefficient has been obtained from the transmittance and reflectance spectra of the films by applying the following relationship [40]:

(1) $$\alpha =\frac{1}{t}\ln\left(\frac{T}{{\left(1-R\right)}^{2}}\right)$$

where *t*, *T*, and *R* are the thickness, transmittance, and reflectance of the sample, respectively. The presence of the slow rising of the absorption coefficient with applying photon energies indicates the amorphous nature of the samples [41]. The estimated values of the absorption edge for the samples were obtained from the intersection of the extrapolation of the linear part of the absorption coefficient to the photon energy axis (see Figure 7). The results are tabulated in Table 1, in which a wide shift of the absorption edge from 4.9 eV for pure PMMA to 2.66 eV for PMMA incorporated with 28 mL GT has been obtained. This reveals the small band gap nature of the doped samples.

### 3.3. Band Gap Study

**Figure 8:**
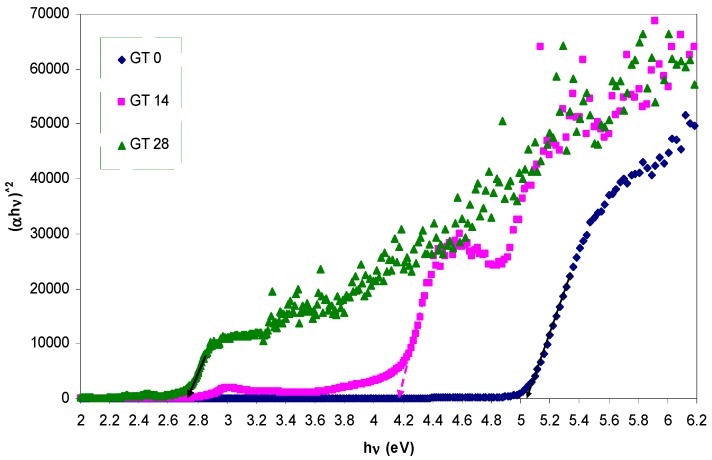
The plots of (α*hυ*)^2^ vs. (*hυ*) for all the samples.

**Figure 9:**
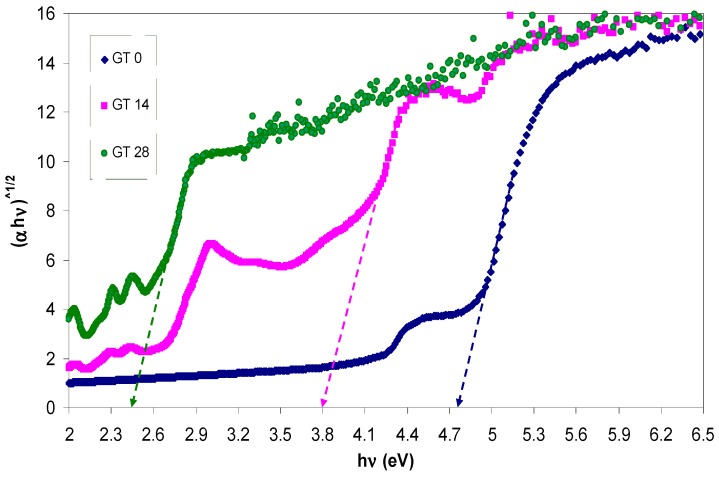
The plots of (α*hυ*)^1/2^ vs. (*hυ*) for all the samples.

**Figure 10:**
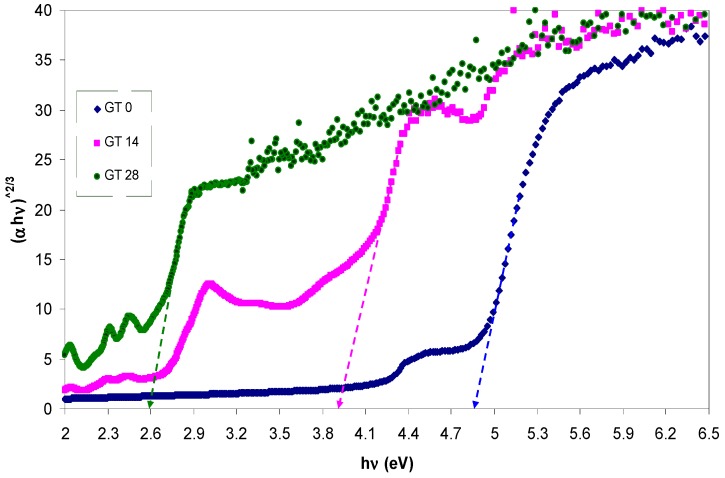
The plots of (α*hυ*)^2/3^ vs. (*hυ*) for all the samples.

**Figure 11:**
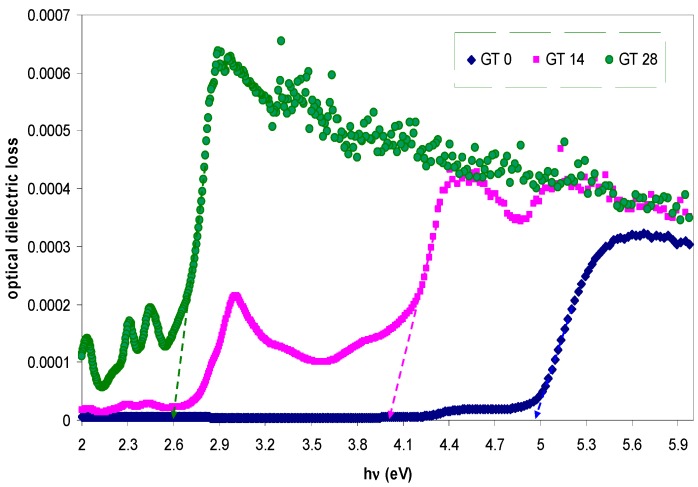
Optical dielectric loss spectra for pure and doped PMMA samples.

The absorption coefficient (α) and the optical band gap (*E_g_*) are expected to be related with each other through the well-known Tauc’s relationship, given by [42,43]:

(2) $$\alpha hv=A{\left(hv-E_{g}\right)}^{\gamma }$$

where *A* is an energy-independent constant and *E_g_* is the optical band gap. Here, the optical band gap energy can be determined by applying Equation (2) to the observed UV–VIS spectra of the samples. Furthermore, the nature of the electronic transition can be determined by specifying the value of γ. For direct transitions, γ takes the values 1/2 or 3/2, whereas γ is equal to 2 or 3 for indirect transitions based on whether they are allowed or forbidden, respectively [44]. In general, insulators/semiconductors are classified into two types of materials: direct and indirect band gaps. In the direct band gap materials, the valance band maximum (VBM) and the conduction band minimum (CBM) coincide at the same zero crystal momentum point (i.e., wave vector *k* = 0) [45]. In this case, γ takes the value of 1/2. In some materials, when the quantum selection rule does not allow the direct transition between the VBM and CBM, the transition is called forbidden direct transition and γ = 3/2. Indirect electron transition occurs when the VBM and the CBM do not lie at same wave vector. In this case, absorption or emission of phonon energy will always be associated to the electron transition from VB to CB with a right magnitude of crystal momentum [46]. To accurately estimate the energy band gap, from the plots of (α*hυ*)^1/γ^ versus the photon energy *hυ*, it is necessary to extrapolate the linear portion of the curve to intersect the photon energy axis (*x*-axis) as shown in Figure 8, Figure 9 and Figure 10. As a consequence, it is difficult to decide the dominant type of electronic transition in the samples. Earlier studies revealed that the value of γ can be achieved by using an analytical differentiation method, which was generally found to be an imprecise method [43,47]. For this purpose, d(ln(α))/d(*hυ*) versus photon energy (*hυ*) was plotted and a maximum peak was achieved. Furthermore, a perpendicular line from the maximum peak to the photon energy axis is drawn to obtain the *E_g_* value. The value of γ was then estimated from the slope of the ln(α*hυ*) versus ln(*hυ* − *E_g_*) curve. This procedure needs considerable time and is not a precise method [43,47]. In this work, optical dielectric loss and Tauc’s model were used to estimate the optical band gap and the electronic transition types, respectively. This is related to the fact that the optical dielectric function hardly depends on materials band structure. At the same time, investigations of the optical dielectric function using UV–VIS spectroscopy are also found to be considerably useful in predicting the overall band structure of the materials [48]. Recent studies have confirmed that the imaginary part of the optical dielectric function, ε″, can mainly be used to describe the electronic transition between occupied and unoccupied states [49,50,51]. The optical dielectric loss spectra obtained for pure and doped PMMA samples are shown in Figure 11. It can be seen that all the samples exhibit a linear behavior at higher photon energies. The imaginary part is seen to be related to the absorption coefficient [52]. It is clear that the optical band gap achieved from optical dielectric loss (see Figure 11) is almost equal to those estimated from Tauc’s model (see Figure 10) for the doped samples. On the other hand, for the pure PMMA sample, the optical band gap estimated from Tauc’s model (see Figure 8) is found to be approximately 5.04 eV, which is considerably close to that achieved (4.97 eV) from the optical dielectric loss plot (see Figure 11). Thus, the type of electronic transition is the allowed direct transition for the pure PMMA sample and the forbidden direct transition for the doped samples. Consequently, it is understood from these results that the complex optical dielectric function can be successfully used for studying the band structure and estimation of optical band gaps. Reducing the optical band gap from 5.04 eV for pure PMMA sample to 2.6 eV for the doped PMMA (GT 28) sample reveals that the extracted GT solution can modify the electronic structure of the host PMMA polymer; in particular, the energy states between the valence and conduction bands. Here, the achieved band gap for the dye-doped PMMA (GT 28) sample is found to be smaller than the recently-reported band gap of Alq3 (2.83 eV) and has gained large popularity among researchers due to its wide applications in photo-detectors, photovoltaic cells, flat and flexible colour displays, and organic light-emitting diodes (OLEDs) [53].

### 3.4. Urbach Energy and Materials Structure

**Figure 12:**
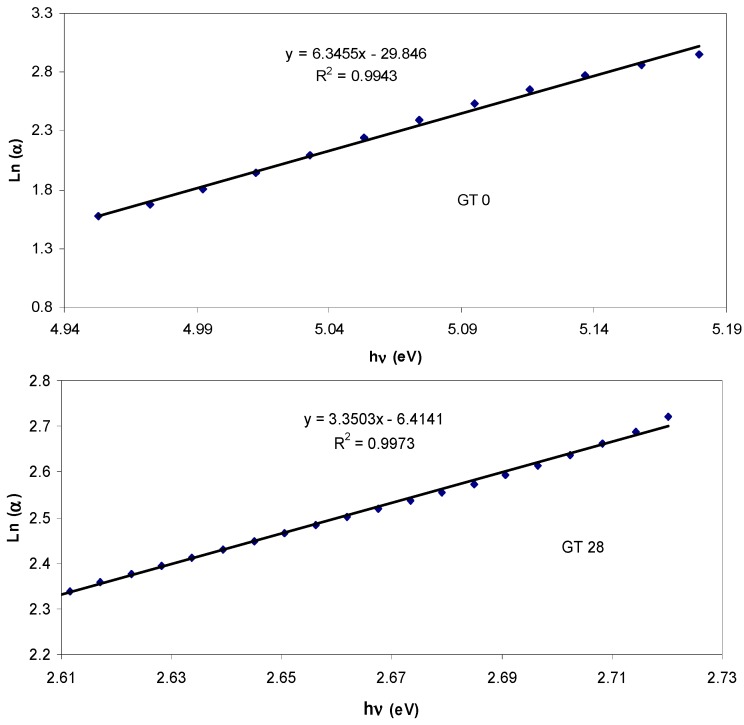
Urbach plot for pure PMMA (GT 0) and PMMA doped (GT 28) samples.

**Figure 13:**
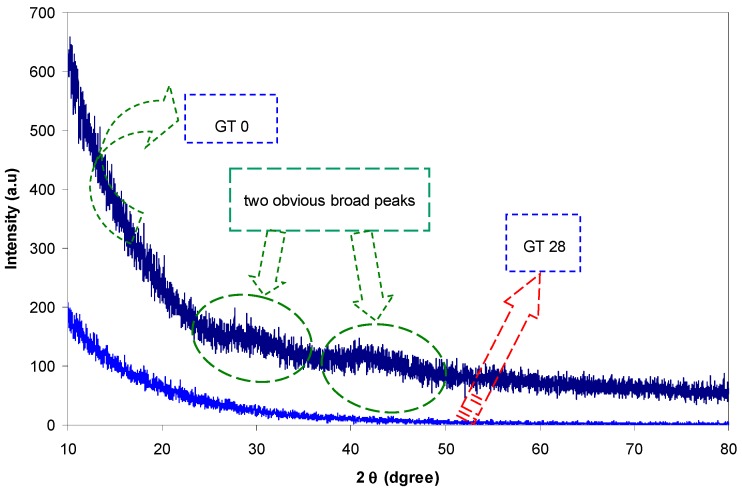
XRD pattern of pure PMMA (GT0) and PMMA doped sample (GT28).

It was established that Urbach energy can be used to investigate the structure of polymeric materials through the detection of the defect level within the forbidden band gap [36]. The Urbach tail width was estimated through the following relation [54,55]:

α_(ω)_*=* α_o_*exp*(*hυ*/*E_t_*) (3)

where α_o_ is constant and *E_t_* is the Urbach tail, which refers to the band tails width of the localized states. One can determine *E_t_* from the reciprocal of the slope of the straight lines obtained from the plots of ln(α) vs. photon energy *hυ* (see Figure 12). The determined value of *E_t_* for the pure PMMA sample is found to be 157 meV, while it increased to 298 meV for the doped PMMA sample (GT 28). This increase of Urbach energy can be indirectly attributed to the increase of the amorphous nature within the dye-doped PMMA samples. The larger energy tails indicate the creation of disorder and imperfection in the band structure of the host material [56]. Prasher et al. have also confirmed that the increase of Urbach energy is an indication of the increase of the amorphous portion [57]. Figure 13 shows the XRD pattern of pure (GT 0) and dye-doped (GT 28) PMMA samples. It is evident from the figure that the PMMA polymer exhibits two broad peaks. The broad peaks appearing around 2θ = 30° and 2θ = 43° reveals the amorphous structure of the pure PMMA polymer [58]. The disappearance of the broad peak in the GT 28 sample reveals the amorphousness of the sample. From the Urbach energy study and XRD analysis, it is understood that the structure of the materials and the optical electronic properties are strongly correlated. The XRD results confirmed the fact that the samples are transferred to complete amorphous phase material after the addition of the extracted GT solution. The achieved Urbach energy values have strongly supported the XRD results. 

## 4. Conclusions

In this work, FTIR spectroscopy was used to investigate the miscibility of green tea (GT) dye and PMMA polymer. In the FTIR spectra of GT extract, obvious absorption bands at 3401 cm^−1^, 1628 cm^−1^, and 1029 cm^−1^, corresponding to O–H/N–H, C=O, and C–O groups were observed, respectively. The FTIR bands shifting and intensity reduction in the doped PMMA sample confirm the complex formation of the host PMMA polymer with the GT dye. The results of this study were promising and revealed the possibility of modification of the insulating wide band gap PMMA polymer to a conjugated small band gap PMMA by addition of extracted GT solution, which is an environmentally friendly material. The absorption edge was found to be 4.9 eV for the pure PMMA and shifted to 2.61 eV for the dye-doped PMMA (GT 28) sample. This reveals that the wide band gap of PMMA was reduced to a narrow energy band gap. Such a noticeable decrease in the optical band gap of PMMA upon the addition of extracted GT solution makes it possible to consider this work as a base to modify other polar polymers to meet our needs. Modified polar polymers with a small band gap and good film formation are crucial for solving the problems, such as lifetime, cost, and flexibility associated with conjugated polymers. The Urbach energy was found to increase from 187 meV for pure PMMA to 298 meV for the dye-doped PMMA (GT 28) sample. This increase was attributed to the dominant of amorphous phase in the dye-doped PMMA samples as supported by XRD results. 

## Figures and Tables

**Table 1:** The absorption edge, optical band gap (from Tauc’s model and ε″ vs. *hυ*) for all the samples.

Sample designation	Absorption edge (eV)	Optical bandgap from Tauc’s model (eV)	Optical bandgap from ε″ vs. *hυ*
GT 0	4.9	5.04, γ = 1/2	4.97
GT 14	3.97	3.94, γ = 3/2	3.97
GT 28	2.66	2.6, γ = 3/2	2.6

## References

[1] Gaur S.S., Ghawana K., Tripathi K.N. (2005). Fabrication and characterization of dye-doped polymeric mode filter. Opt. Quantum Electron..

[2] Chandrahalim H., Fan X. (2015). Reconfigurable solid-state dye-doped polymer ring resonator lasers. Sci. Rep..

[3] Ishchenko A.A. (2008). Photonics and molecular design of dye-doped polymers for modern light-sensitive materials. Pure Appl. Chem..

[4] Maji P., Choudhary R.B., Majhi M. (2016). Structural, optical and dielectric properties of ZrO_2_ reinforced polymeric nanocomposite films of polymethylmethacrylate (PMMA). Optik.

[5] Alsawafta M., Badilescu S., Paneri A., Truong V.V., Packirisamy M. (2011). Gold-poly(methyl methacrylate) nanocomposite films for plasmonic biosensing applications. Polymers.

[6] Hamdy M.S., AlFaify S., Al-Hajry A., Yahia I.S. (2016). Optical constants, photo-stability and photo-degradation of MB/PMMA thin films for UV sensors. Optik.

[7] Ebnalwaleda A.A., Thabet A. (2016). Controlling the optical constants of PVC nanocomposite films for optoelectronic applications. Synth. Met..

[8] Hassan H.E., Refat M.S., Sharshar T. (2016). Optical and positron annihilation spectroscopic studies on PMMA polymer doped by rhodamine B/chloranilic acid charge transfer complex: Special relevance to the effect of γ-rays irradiation. Spectrochim. Acta A.

[9] Enculescu M., Matei E. (2016). Influence of metallic and semiconducting nanostructures on the optical properties of dye-doped polymer thin films. Thin Solid Films.

[10] Fleischmann C., Lievenbrück M., Ritter H. (2015). Polymers and dyes: Developments and applications. Polymers.

[11] Ishchenko A. (2002). Molecular engineering of dye-doped polymers for optoelectronics. Polym. Adv. Technol..

[12] Sun X., Chang F., Gai K. (2016). Optoelectronic fast response properties of PQ/PMMA polymer. Mater. Today Proc..

[13] Lee L.S., Kim S.H., Kim Y.B., Kim Y.C. (2014). Quantitative analysis of major constituents in green tea with different plucking periods and their antioxidant activity. Molecules.

[14] Reto M., Figueira M.E., Filipe H.M., Almeida C.M. (2007). Chemical composition of green tea (*Camellia sinensis*) infusions commercialized in Portugal. Plant Foods Hum. Nutr..

[15] Lee Y.H., Hwang E.K., Kim H.D. (2009). Colorimetric assay and antibacterial activity of cotton, silk, and wool fabrics dyed with peony, pomegranate, clove, coptischinenis and gallnut extracts. Materials.

[16] Hwang E.K., Lee Y.H., Kim H.D. (2008). Dyeing, fastness, and deodorizing properties of cotton, silk, and wool fabrics dyed with gardenia, coffee sludge, *Cassia tora*. L., and pomegranate extracts. Fibers Polym..

[17] Loo Y.Y., Chieng B.W., Nishibuchi M., Radu S. (2012). Synthesis of silver nanoparticles by using tea leaf extract from *Camellia sinensis*. Int. J. Nanomed..

[18] Senthilkumar S.R., Sivakumar T. (2014). Green tea (*Camellia sinensis*) mediated synthesis of zinc oxide (ZnO) nanoparticles and studies on their antimicrobial activities. Int. J. Pharm. Pharm. Sci..

[19] Dubey S.P., Sillanpaa M., Varma R.S. (2017). Reduction of hexavalent chromium using *Sorbaria sorbifolia* aqueous leaf extract. Appl. Sci..

[20] Huang L., Weng X., Chen Z., Megharaj M., Naidu R. (2014). Synthesis of iron-based nanoparticles using oolong tea extract for thedegradation of malachite green. Spectrochim. Acta A.

[21] Weng X., Huang L., Chen Z., Megharaj M., Naidu R. (2013). Synthesis of iron-based nanoparticles by green tea extract and theirdegradation of malachite. Ind. Crop. Prod..

[22] Ahmed R.M. (2009). Optical study on poly(methyl methacrylate)/poly(vinyl acetate) blends. Int. J. Photoenergy.

[23] Kalsi P.S. (2004). Spectroscopy of Organic Compounds.

[24] Balamurugan A., Kannan S., Selvaraj V., Rajeswari S. (2004). Development and spectral characterization of poly(methyl methacrylate)/hydroxyapatite composite for biomedical applications. Trends Biomater. Artif. Organs.

[25] Aziz S.B., Abidin Z.H.Z. (2013). Electrical conduction mechanism in solid polymer electrolytes: New concepts to Arrhenius equation. J. Soft Matter.

[26] Wei D., Sun W., Qian W., Ye Y., Ma X. (2009). The synthesis of chitosan-based silver nanoparticles and their antibacterial activity. Carbohydr. Res..

[27] Soman V.V., Kelkar D.S. (2009). FTIR studies of doped PMMA—PVC blend system. Macromol. Symp..

[28] Kumar R., Ali S.A., Mahur A.K., Virk H.S., Singh F., Khan S.A., Avasthi D.K., Prasad R. (2008). Study of optical band gap and carbonaceous clusters in swift heavy ion irradiated polymers with UV–Vis spectroscopy. Nucl. Instrum. Methods Phys. Res. B.

[29] Ingle J.D., Crouch S.R. (1988). Spectrochemical Analysis.

[30] Srivastava A., Singh V., Aggarwal P., Schneeweiss F., Scherer U.W., Friedrich W. (2010). Optical studies of insulating polymers for radiation dose monitoring. Indian J. Pure Appl. Phys..

[31] Marzuki A., Suryanti V., Virgynia A. (2017). Spectroscopic study of green tea (*Camellia sinensis*) leaves extraction. IOP Conf. Ser. Mater. Sci. Eng..

[32] Kumar K.R.P., Murali M.G., Udayakumar D. (2014). Synthesis and study of optical properties of linear and hyperbranched conjugated polymers containing thiophene and riphenylamine units. Des. Monomers Polym..

[33] Koyuncu F.B., Sefer E., Koyuncu S., Ozdemir E. (2011). A new low band gap electrochromic polymer containing 2,5-bis-dithienyl-1H-pyrrole and 2,1,3-benzoselenadiazole moiety with high contrast ratio. Polymer.

[34] Noelia L.G., Roberto R.G., Patricia P.B., Vidal J.L.M., Frenich A.G. (2015). Identification and quantification of phytochemicals in nutraceutical products from green tea by UHPLC–Orbitrap-MS. Food Chem..

[35] Pasrija D., Anandharamakrishnan C. (2015). Techniques for extraction of green tea polyphenols: A review. Food Bioprocess Technol..

[36] Aziz S.B. (2016). Modifying poly(vinyl alcohol) (PVA) from insulator to small band gap polymer: A novel approach for organic solar cells and optoelectronic devices. J. Electron. Mater..

[37] Arslan M., Atak F.B., Yakuphanoglu F. (2007). Synthesis and refractive index dispersion properties of the *N*,*N*′,*N*′′-trinaphthylmethyl melamine–DDQ complex thin film. Opt. Mater..

[38] Calvert P. (2012). Optical Properties of Polymer Composites. Wiley Encyclopedia of Composites.

[39] Yakuphanoglu F., Sekerci M., Ozturk O.F. (2004). The determination of the optical constants of Cu(II) compound having 1-chloro-2,3-*o*-cyclohexylidinepropane thin film. Opt. Commun..

[40] Yakuphanoglu F., Cukurovali A., Yilmaz I. (2005). Refractive index and optical absorption properties of the complexes of a cyclobutane containing thiazolylhydrazone ligand. Opt. Mater..

[41] Yakuphanoglu F., Viswanathan C. (2007). Electrical conductivity and single oscillator model properties of amorphous CuSe semiconductor thin film. J. Non-Cryst. Solids.

[42] Bhajantri R.F., Ravindrachary V., Harisha A., Crasta V., Nayak S.P., Poojary B. (2006). Microstructural studies on BaCl_2_ doped poly(vinyl alcohol). Polymer.

[43] Yakuphanoglu F., Arslan M. (2004). Determination of electrical conduction mechanism and optical band gap of a new charge transfer complex: TCNQ-PANT. Solid State Commun..

[44] Abdullah O.G., Aziz S.B., Omer K.M., Salih Y.M. (2015). Reducing the optical band gap of polyvinyl alcohol (PVA) based nanocomposite. J. Mater. Sci. Mater. Electron..

[45] Mohan V.M., Bhargav P.B., Raja V., Sharma A.K., Rao V.V.R.N. (2007). Optical and electrical properties of pure and doped PEO polymer electrolyte films. Soft Mater..

[46] Kumar K.K., Ravi M., Pavani Y., Bhavani S., Sharma A.K., Rao V.V.R.N. (2011). Investigations on the effect of complexation of NaF salt with polymer blend (PEO/PVP) electrolytes on ionic conductivity and optical energy band gaps. Phys. B Condens. Matter.

[47] Yakuphanoglu F., Arslan M. (2004). The fundamental absorption edge and optical constants of some charge transfer compounds. Opt. Mater..

[48] Kittel C. (2005). Introduction to Solid State Physics.

[49] Aziz S.B., Rasheed M.A., Ahmed H.M. (2017). Synthesis of polymer nanocomposites based on [methyl cellulose]_(1-*x*)_:(CuS)*_x_* (0.02 M ≤ *x* ≤ 0.08 M) with desired optical band gaps. Polymers.

[50] Aziz S.B., Abdullah O.G., Rasheed M.A. (2017). A Novel polymer composite with a small optical bandgap: New approaches for photonics and optoelectronics. J. Appl. Polym. Sci..

[51] Aziz S.B., Abdullah O.G., Hussein A.M., Abdulwahid R.T., Rasheed M.A., Ahmed H.M., Abdalqadir S.W., Mohammed A.R. (2017). Optical properties of pure and doped PVA:PEO based solid polymer blend electrolytes: Two methods for band gap study. J. Mater. Sci. Mater. Electron..

[52] Patterson J.D., Bailey B.C. (2007). Solid State Physics: Introduction to the Theory.

[53] El-Nahass M.M., Farid A.M., Atta A.A. (2010). Structural and optical properties of Tris(8-hydroxyquinoline) aluminum (III) (Alq_3_) thermal evaporated thin films. J. Alloys Compd..

[54] Ahmad F., Sheha E. (2013). Preparation and physical properties of (PVA)_0.7_(NaBr)_0.3_(H_3_PO_4_)_xM_ solid acid membrane for phosphoric acid—Fuel cells. J. Adv. Res..

[55] Sheha E., Khoder H., Shanap T.S., El-Shaarawy M.G., El Mansy M.K. (2012). Structure, dielectric and optical properties of p-type (PVA/CuI) nanocomposite polymer electrolyte for photovoltaic cells. Optik.

[56] Aziz S.B., Ahmed H.M., Hussein A.M., Fathulla A.B., Wsw R.M., Hussein R.T. (2015). Tuning the absorption of ultraviolet spectra and optical parameters of aluminum doped PVA based solid polymer composites. J. Mater. Sci. Mater. Electron..

[57] Prasher S., Kumar M., Singh S. (2014). Electrical and optical properties of O^6+^ion beam–irradiated polymers. Int. J. Polym. Anal. Charact..

[58] Aziz S.B., Abdulwahid R.T., Rasul H.A., Ahmed H.M. (2016). In situ synthesis of CuS nanoparticle with a distinguishable SPR peak in NIR region. J. Mater. Sci. Mater. Electron..

